# Colorectal Cancer Organoid Model Reveals the Mechanisms of Irinotecan Resistance at Single‐Cell Resolution

**DOI:** 10.1002/cam4.71550

**Published:** 2026-02-12

**Authors:** Yi Pan, Lin Chen, Yuqing Hu, Jie Chang, Xifeng Xu, ShuoChen Xu, YiWen Li, Jinlin Du, JianPing Wang, Wenxia Xu

**Affiliations:** ^1^ Central Laboratory and Precision Medicine Center, Affiliated Jinhua Hospital Zhejiang University School of Medicine Jinhua Zhejiang Province China; ^2^ Jinhua Key Laboratory of Cancer Nutrition and Metabolism Research, Affiliated Jinhua Hospital Zhejiang University School of Medicine Jinhua Zhejiang Province China; ^3^ Department of Oncology, Affiliated Jinhua Hospital Zhejiang University School of Medicine Jinhua Zhejiang Province China; ^4^ Department of Colorectal Surgery, Affiliated Jinhua Hospital Zhejiang University School of Medicine Jinhua Zhejiang Province China

**Keywords:** irinotecan resistance, lipid metabolism, notch signaling, patient‐derived colorectal cancer organoids, single‐cell RNA sequencing

## Abstract

**Objective:**

Irinotecan, a standard therapeutic agent for metastatic colorectal cancer (mCRC), often faces significant limitations due to drug resistance, with treatment failure observed in approximately 30%–50% of patients, leading to poor clinical outcomes. This study aims to systematically elucidate the molecular mechanisms underlying irinotecan resistance in colorectal cancer (CRC) by constructing patient‐derived organoid (PDO) models combined with single‐cell transcriptomics technology.

**Methods:**

PDO models were successfully established from irinotecan‐resistant and sensitive CRC patients. Single‐cell RNA sequencing (scRNA‐seq) was performed on the organoids, analyzing the transcriptomic heterogeneity of 12,360 high‐quality cells. Gene Set Variation Analysis (GSVA), transcriptional regulatory networks, and cell communication networks were employed to dissect the resistance mechanisms.

**Results:**

Drug sensitivity assays demonstrated that the IC_50_ value of irinotecan in CRC5 was significantly higher than that in CRC11, which was entirely consistent with their respective clinical phenotypes. Single‐cell sequencing identified CRC5‐specific drug‐resistant cell clusters, Cluster 1 and Cluster 6. Cluster 1 (MARCKSL1+) was characterized by the activation of the Wnt signaling pathway and extracellular matrix (ECM) remodeling, which collectively contributed to the maintenance of stem cell‐like properties, while Cluster 6 (AKR1C3+) exhibited significant enrichment in lipid metabolism and the Notch signaling pathway.

**Conclusion:**

This study integrates PDO models with single‐cell transcriptomics technology to reveal key cell subpopulations and molecular mechanisms underlying irinotecan resistance. The core mechanisms driving resistance involve the activation of Wnt signaling and the synergistic effect of lipid metabolism‐Notch pathways. Cluster 1 and Cluster 6 are identified as potential therapeutic targets, providing a theoretical basis for developing combination therapies targeting cancer stem cells or the metabolic microenvironment.

## 1 Introduction

Colorectal cancer (CRC), ranking as the third most common malignant tumor and the second leading cause of cancer‐related death globally, witnesses over 1.9 million new cases and 930,000 deaths annually [1, 2]. In the systemic treatment of advanced CRC, irinotecan, a topoisomerase I inhibitor, in combination with 5‐fluorouracil (5‐FU) forms the standard chemotherapy regimen (FOLFIRI), significantly prolonging patient survival [3, 4, 5, 6]. However, chemoresistance limits its clinical efficacy, with 30%–50% of patients experiencing therapeutic failure due to multifactorial mechanisms, including aberrant drug metabolism, efflux pump activation, and tumor microenvironment remodeling [7, 8, 9, 10, 11]. Notably, irinotecan requires carboxylesterase‐mediated conversion to its active metabolite SN‐38 (7‐ethyl‐10‐hydroxycamptothecin), whose cytotoxic effects are counteracted by UGT1A1‐driven glucuronidation [12]. Dysregulation of this metabolic pathway has been strongly associated with resistance [13]. However, the precise molecular orchestration underlying irinotecan resistance remains poorly delineated.

Traditional two‐dimensional (2D) cell models fail to recapitulate tumor heterogeneity and microenvironmental interactions, limiting their utility in resistance studies [14]. Recent advances in organoid technology have enabled the establishment of three‐dimensional (3D) culture systems that preserve primary tumors' genetic, phenotypic, and stromal heterogeneity, offering clinically relevant platforms for drug response evaluation [15, 16, 17]. Specifically, CRC‐derived organoids retain patient‐specific mutational profiles and drug sensitivity patterns, demonstrating unique advantages in personalized medicine and drug discovery.

Single‐cell RNA sequencing (scRNA‐seq) provides unprecedented resolution for dissecting cellular heterogeneity and intercellular communication networks [18, 19, 20]. Unlike bulk omics approaches, this technology enables precise identification of gene expression variations across individual cells, uncovering distinct cellular states and subtypes critical for understanding tumor evolution and therapy resistance [21]. Importantly, scRNA‐seq is uniquely positioned to resolve the transient transcriptional programs driving irinotecan resistance—such as the emergence of ABCG2+ drug‐tolerant persister cells—that are often obscured in bulk analyses due to transcriptional averaging [22].

This study integrates organoid models with single‐cell sequencing technology to systematically investigate how tumor cells evade irinotecan treatment by altering gene expression profiles and intercellular communication networks. Our findings demonstrate that irinotecan resistance in CRC is associated with aberrant activation of Wnt signaling and functional synergy between lipid metabolic reprogramming and Notch pathway activity. These insights advance our understanding of irinotecan chemoresistance mechanisms in CRC and propose potential therapeutic strategies to counteract treatment resistance.

## 2 Materials and Methods

### 2.1 Human Specimens

This study was approved by the Ethics Committee of Jinhua Central Hospital ((Research) 2021—Ethical Review—257). Primary CRC biopsy specimens were obtained from the Department of Colorectal Surgery, Affiliated Jinhua Hospital, Zhejiang University School of Medicine. Clinical information from patients who received NACR treatment was collected from medical records, including MRI images and pathological tumor regression grade (TRG) data. Informed consent was obtained from all participants. The samples should be stored in Advanced DMEM/F—12 medium (Gibco, 12634028) at 4°C and processed within 2 h.

### 2.2 Culture of CRC Patient‐Derived Organoids (CRC‐PDOs)

Colon cancer patient‐derived organoids (PDOs) were established in previous studies and evaluated for morphological and genomic characteristics. Methods of organoid culture for colon cancer have been described in previous studies [23]. In simple terms, the cell pellet was resuspended in an appropriate volume of serum‐free DMEM medium and mixed with Cultrex Reduced Growth Factor Basement Membrane Extract, Type 2 (R&D, 3533‐010‐02) at a 1:1 volume ratio. The mixture was then seeded at 20 μL per well into a 48‐well plate to establish a three‐dimensional culture system. Organoids were maintained in a specialized CRC medium (Shanghai Womiao Biotechnology Co. LTD, WMH‐03). Organoid clusters were dissociated for passaging using 400 μL of TrypLE Express enzyme (Gibco) for 10–15 min, followed by gentle pipetting (1–3 min) to obtain single‐cell suspensions. The cells were subsequently re‐embedded in fresh Matrigel for expansion culture. The PDO medium was refreshed every 3 days, and morphological changes of the organoids were monitored throughout the culture period.

### 2.3 Drug Assay of CRC‐PDOs

PDOs were harvested and dissociated into single cells following the passaging procedure described above. Cell pellets were resuspended in 500 μL of PDO medium WMH‐03. Cells were counted with the Countess Automated Cell Counter (Thermo Fisher Scientific). 80 μL of cell suspension containing 3000 cells was seeded in Ultra‐Low Attachment Black 96‐Well Plates with Clear Flat Bottom (Shanghai, ByoGold). There are 3 duplicate wells in each group. After 3 days, 20 μL of complete culture medium containing SN‐38 (Selleck, #S4908) was added. Following 72 h of treatment, organoid morphology was captured under a microscope. The optical density at 450 nm was measured following 1‐h incubation with CCK‐8 reagent (Beyotime, #C0042). For combination therapy treatment, organoids were cultured in fresh PDO medium containing SN‐38, IWP‐2 (TargetMol, #T2702), DAPT (TargetMol, #T6202), MF‐438 (TargetMol, #T16068), Vorapaxar (TargetMol, #T7013) or the combination of them. The concentration of IWP‐2 [24, 25], DAPT [26], MF‐438 [27], and Vorapaxar [28] selected for the viability assays was determined based on prior established evidence.

### 2.4 Single‐Cell RNA Sequencing Data Processing

Single‐cell RNA sequencing data for both CRC5 and CRC11 samples were generated in a single, continuous experimental run to minimize technical batch effects. The data were processed using Cellranger (version 6.0.2), where transcript reads from 10× Genomics were aligned to the GRCh38 human genome reference using default parameters. Cells with exceptionally high mitochondrial gene expression (> 20%) or high ribosomal gene expression (> 30%) were excluded from further analysis. The Scanpy package (version: 1.9.3) [29] was then used for subsequent normalization and regression. Specifically, the raw UMI counts were normalized per cell, and the data were scaled with regression against the total UMI counts per sample and the percentage of mitochondrial gene expression to remove these sources of technical variation. The processed expression matrices from the two samples were merged, and clustering was performed using the Seurat package (version 4.0.4). The merged expression matrix was then reduced to two dimensions using UMAP (Uniform Manifold Approximation and Projection) for better visualization and identification of distinct cell populations [30]. The CellCycleScoring function in the Seurat package (version 4.0.4) was employed to evaluate cell cycle variation across all cells [31].

### 2.5 Differential Gene Expression and Gene Enrichment Analysis (DEG)

Differentially expressed genes (DEGs) for each cluster were identified using the FindAllMarkers function of the Seurat package (version 4.0.4) [32]. Significant differential expression was determined by the Wilcoxon Rank‐Sum test, and DEGs were selected for subsequent analysis (average Log_2_FoldChange ≥ 0.25). Gene Ontology (GO) and Kyoto Encyclopedia of Genes and Genomes (KEGG) enrichment analyses were performed using the clusterProfiler package (version 3.18.1) in R [33], and results were visualized using the ggplot2 package (version 3.3.5).

### 2.6 Non‐Negative Matrix Factorization (NMF) and Gene Enrichment

Non‐negative Matrix Factorization (NMF) was used to compute representative gene modules for each cluster. Gene enrichment analysis was performed on these modules using the clusterProfiler package (version 3.18.1) in R, and the results were visualized using the ggplot2 package (version 3.3.5).

### 2.7 Gene Set Variation Analysis (GSVA)

Gene sets related to proliferation, stemness, invasion, and metabolism were downloaded from the Molecular Signatures Database (MSigDB). After normalizing the single‐cell expression matrix, Gene Set Variation Analysis (GSVA) was conducted to perform functional enrichment analysis on the single‐cell RNA sequencing (scRNA‐seq) data, aiming to reveal functional differences between various cell subpopulations at the gene set level [34].

### 2.8 Pseudotime Analysis

To explore the dynamic changes of cells during differentiation and analyze gene expression variations across different states, pseudotime analysis was performed. Initially, we used the CytoTRACE R package (version 0.3.3) to distinguish cell states between the two samples and assess the differentiation states of different subpopulations. Subsequently, we applied the Monocle2 R package (version 2.18.0) for pseudotime analysis, using differentiation states determined by CytoTRACE to explore relationships between subpopulations [35]. The top 10 differential genes in pseudotime were visualized using the differentialGeneTest and plot_pseudotime_heatmap functions. Additionally, we used the Venn R package (version 1.10) to identify overlapping genes between the top 1000 pseudotime genes (ranked by *p*‐value) from both samples, followed by enrichment analysis and visualization.

### 2.9 Cell–Cell Communication Analysis

To investigate intercellular signaling and reveal communication networks between different cell populations, we used the CellChat R package (version 1.1.3) to compute relevant cell–cell communications [36]. This analysis helped identify active signaling pathways and potential key regulatory nodes by studying specific signaling pathways and cell–cell interactions. Additionally, ligand‐receptor analysis was performed to identify and quantify ligand‐receptor interactions between different cell types, thus uncovering the cell communication network.

### 2.10 Gene Regulatory Network Analysis and Identification of Hub Genes

To identify key regulatory factors potentially influencing drug response, we performed gene regulatory network (GRN) analysis and transcription factor enrichment analysis for each cluster using the SCENIC R package (version 1.1.0) [37]. AUCell was employed to compute the activity and regulatory specificity scores (RSS) of each regulon within individual cells. Hub genes in the 8 identified GRNs were identified using CytoHubba (version 3.8.2) in Cytoscape. Using the MCC method, centrality measures were used to assess node importance, with the top 10 centrality‐ranked nodes in each network designated as hub genes. Gene enrichment analysis for each GRN was performed using the clusterProfiler package (version 3.18.1), and overlap between clusters' transcription factors and regulons was visualized using the Venn R package (version 1.10). The activity of each regulon within the clusters was computed with AUCell and visualized using the pheatmap R package (version 1.0.12).

### 2.11 Statistical Analysis

Numerical data are expressed as the mean ± SEM. Statistical significance between means was evaluated using the Mann–Whitney test. A *p*‐value ≤ 0.05 was considered statistically significant.

## 3 Results

### 3.1 Clinical Characteristics of the Patients

To investigate the mechanisms of irinotecan resistance in CRC treatment, this study selected two patients with typical and representative clinical features. The detailed clinical characteristics of these patients are shown in Table 1. Patient CRC5 was a 69‐year‐old male diagnosed with moderately differentiated rectal adenocarcinoma. He resisted irinotecan treatment, with a TNM stage of T3N0M0 and a TRG of G2. His microsatellite status was microsatellite stable (MSS). Patient CRC11 was a 30‐year‐old male diagnosed with moderately differentiated sigmoid colon adenocarcinoma. He showed sensitivity to irinotecan treatment, with a TNM stage of T3N0Mx, TRG of G3, and microsatellite status of MSS. The organoids from these patients' tumor tissues have been reported [23], By comparing the resistance of patient CRC5 and the sensitivity of patient CRC11, these organoids were used for further investigation of the molecular mechanisms underlying irinotecan resistance. This comparison facilitates the identification of key factors contributing to irinotecan resistance in CRC and provides important insights for optimizing clinical treatment strategies.

**TABLE 1:** Clinical characteristics of the patients.

Patient ID	CRC5	CRC11
Cancer type	Moderately differentiated rectal adenocarcinoma	Moderately differentiated sigmoid colon adenocarcinoma
Primary site	Rectum	Sigmoid colon
Histology	Adenocarcinoma	Adenocarcinoma
Irinotecan sensitivity	Resistant	Sensitive
Diagnostic stage	T3N0M0	T3N0Mx
Age	69 years	30 years
Gender	Male	Male
Microsatellite status	MSS	MSS
Sample source	Primary tumor	Primary tumor
TRG level	G2	G3

### 3.2 Drug Sensitivity and Immunohistochemical Analysis of Organoids

**FIGURE 1:**
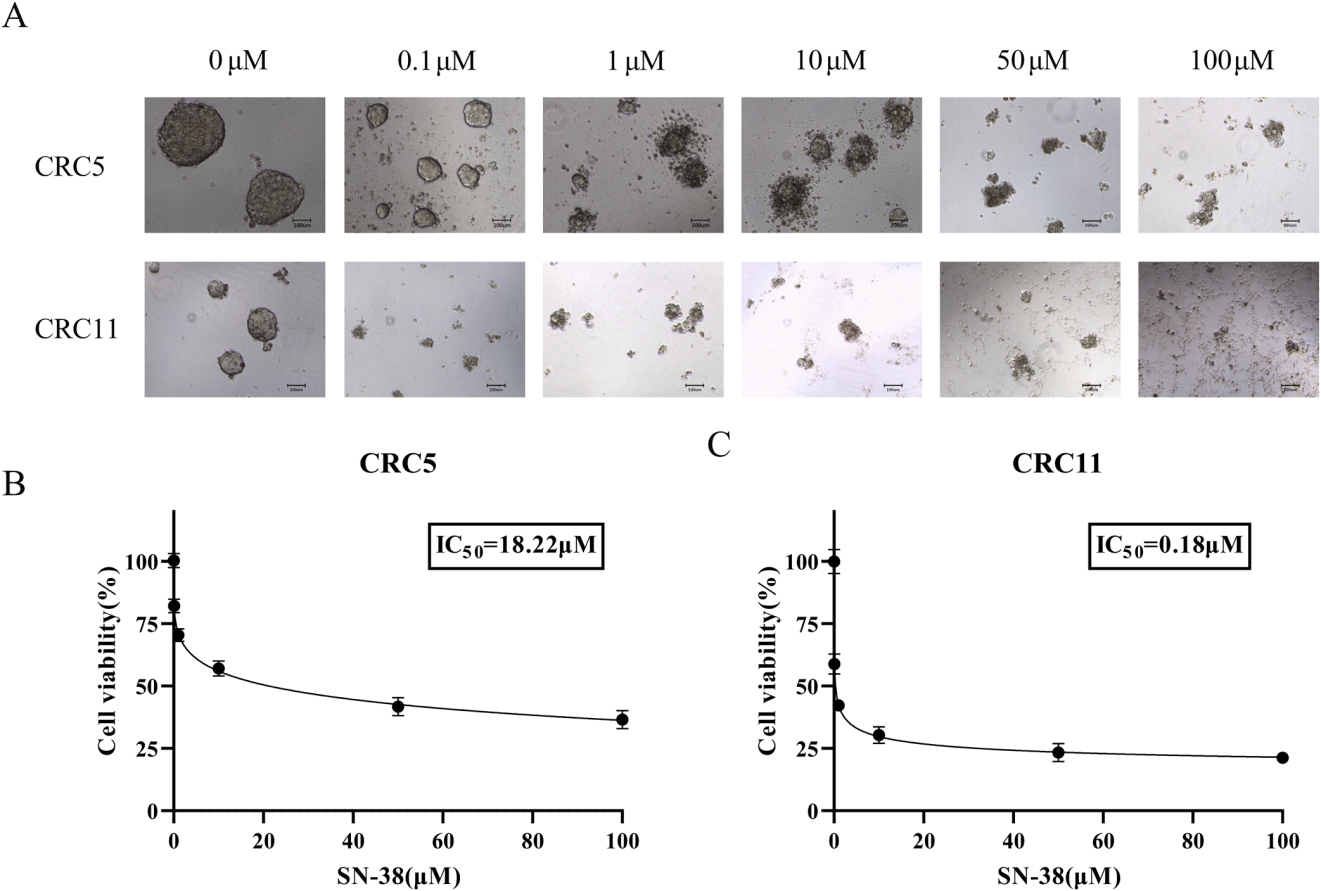
Drug sensitivity results of colorectal cancer organoids. (A) Microscopic images show the morphological changes of CRC5 and CRC11 organoids after treatment with different concentrations of SN‐38 (0 μM, 0.1 μM, 1 μM, 10 μM, 50 μM, and 100 μM). (B–C) The inhibition rate curves which were detected by CCK‐8 kit demonstrated the differential sensitivity of CRC5 and CRC11 organoids to SN‐38.

To evaluate the drug sensitivity characteristics of the established CRC organoid models, we performed SN‐38 sensitivity assays on CRC5 and CRC11 organoids. At the end of the experiment, the organoids were stained with 4% Trypan Blue. Microscopic observation revealed morphological changes in the organoids following treatment with different concentrations of SN‐38 (Figure 1A). In CRC5 organoids, partial dissolution and death of organoid clusters were observed as the SN‐38 concentration increased from 0 μM to 100 μM. However, a portion of the organoid clusters consistently survived, suggesting that CRC5 organoids may possess a certain degree of tolerance to SN‐38. In contrast, CRC11 organoids exhibited dose‐dependent morphological alterations, with increasing disruption and disorganization of cellular structures as SN‐38 concentration increased, indicating a pronounced dose‐dependent response to SN‐38.

To further quantify the sensitivity of the organoids to SN‐38, we measured the inhibition rates and plotted dose–response curves (Figure 1B,C) to determine the half‐maximal inhibitory concentration (IC50) for SN‐38. The results showed that, within 10 μM of SN‐38, the inhibition rate of CRC5 organoids remained consistently low, reaching 50% inhibition at 18.22 μM. In contrast, the inhibition rate of CRC11 organoids increased significantly with rising SN‐38 concentration, with an IC_50_ value of approximately 0.18 μM. The results show that CRC11 was sensitive to SN‐38, while CRC5 was relatively insensitive to SN‐38. These findings are consistent with the morphological observations of the organoids and align with the clinical treatment backgrounds of the patients. These results further validate the reliability and effectiveness of the established CRC organoid models in simulating the drug response characteristics of the primary tumors, providing a robust experimental basis for subsequent in‐depth investigations into the mechanisms of drug resistance in CRC.

### 3.3 Cell Subpopulation Annotation and Differential Analysis

**FIGURE 2:**
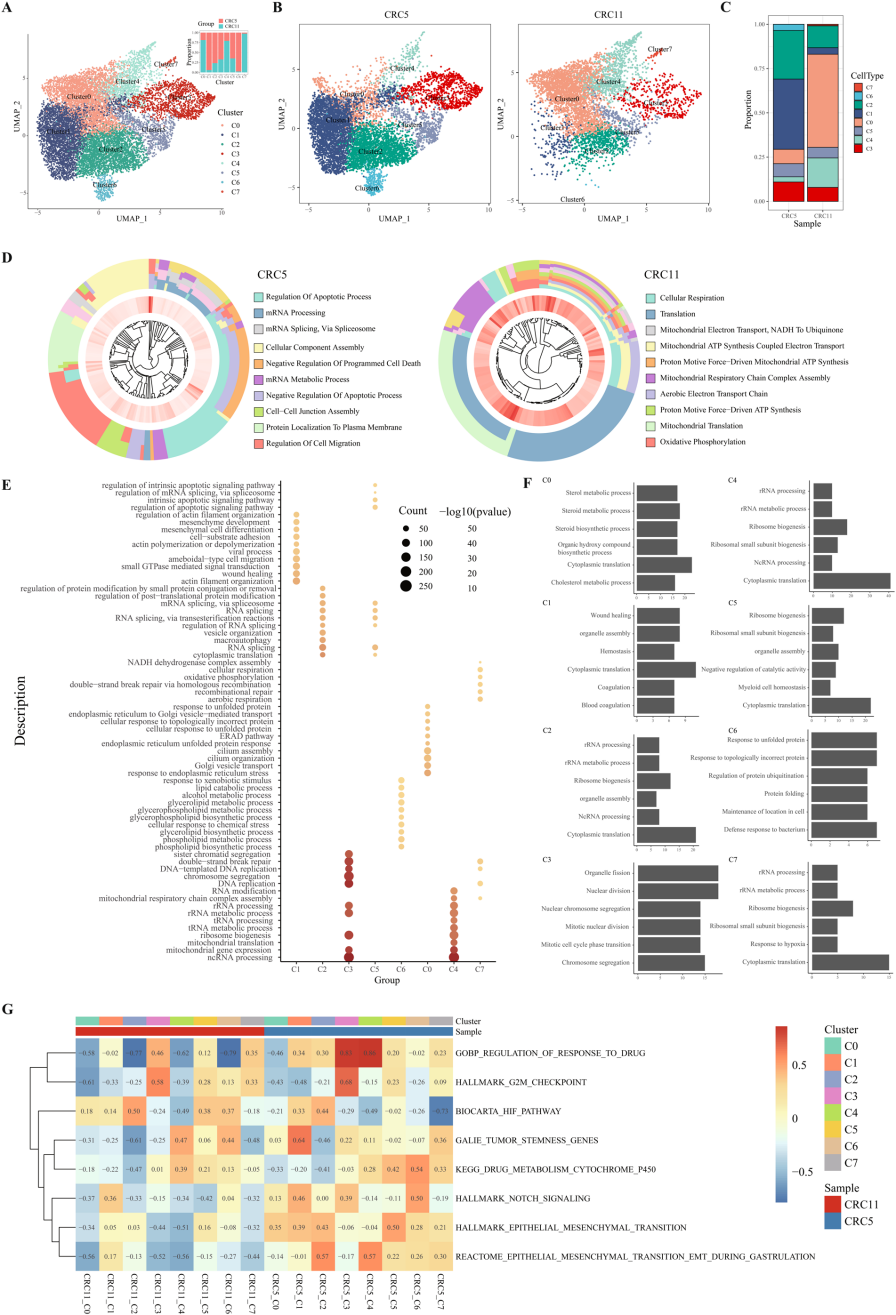
Single‐cell sequencing results of the two organoid models. (A) Overview of clustering and dimensionality reduction using the UMAP method, along with the distribution of cells from each organoid in each cluster. (B) UMAP clustering results for each organoid. (C) Proportional distribution of cells in different clusters between the two organoids. (D) Functional enrichment analysis of differentially expressed genes between the two organoids. (E) GO enrichment analysis of differentially expressed genes within each cluster. (F) Gene module enrichment analysis for each cluster. (G) Gene set variation analysis (GSVA) for each cluster.

To further investigate the molecular mechanisms of irinotecan resistance, we performed single‐cell RNA sequencing on CRC5 and CRC11 organoids, yielding a total of 17,268 cells. After rigorous quality control, 12,360 high‐quality cells were retained for subsequent analysis. Using the UMAP method for clustering and dimensionality reduction, we identified multiple distinct cell clusters (Figure 2A,B). By comparing the distribution of each cluster between the two organoids, we observed significant differences in the proportions of eight clusters between CRC5 and CRC11 (Figure 2C). To pinpoint the cell subpopulations most strongly associated with resistance, we applied quantitative thresholds based on fold‐change abundance. Cluster 1 (marked by MARCKSL1) and Cluster 6 (marked by AKR1C3) emerged as the most salient candidates. Cluster 1 exhibited a greater than 10‐fold increase in CRC5 (2930 cells) compared to CRC11 (182 cells), while Cluster 6 was almost exclusively present in CRC5 (256 cells) and nearly absent in CRC11 (6 cells). Although Clusters 3 and 5 also showed increased abundance in CRC5, the differences were more modest (~2‐fold) and did not meet our predefined thresholds for further investigation. Therefore, we focused subsequent analyses on Clusters 1 and 6 as the core resistant‐associated populations.

To explore the global transcriptional differences between the two organoid models, we conducted Gene Ontology (GO) functional enrichment analysis on the differentially expressed genes in both organoids (Figure 2D). The results revealed that CRC5 was significantly enriched in pathways related to the regulation of apoptosis, mRNA processing and metabolism, RNA splicing, and programmed cell death, indicating that CRC5 may promote resistance through the modulation of apoptosis and RNA metabolism. In contrast, CRC11 was notably enriched in pathways related to cellular respiration, mitochondrial translation, the electron transport chain, and oxidative phosphorylation, suggesting that the sensitive organoid maintains a state of active cellular energy metabolism (Figure 2D).

To independently validate the relevance of these resistance‐associated clusters, we leveraged the publicly available GEO dataset GSE145356. By applying a deconvolution algorithm to this cohort, we demonstrated that Clusters 1 and 6 identified in our study exhibited analogous expression patterns in this independent dataset (Figure S1A–D), thereby reinforcing the biological significance of our findings.

Next, we performed functional enrichment analysis on the differentially expressed genes in each cluster, revealing specific enrichment patterns for each cluster (Figure 2E). Notably, cluster 1 was significantly enriched in coagulation‐related pathways, while cluster 6 exhibited high activity in lipid metabolism. To further investigate the gene modules associated with inter‐cluster differences, we applied Non‐negative Matrix Factorization (NMF) to compute the gene modules for each cluster (Figure 2F). The functional annotations of these modules provided valuable insights into the biological characteristics of the different clusters.

Additionally, we conducted Gene Set Variation Analysis (GSVA) on pathways related to proliferation, stemness, invasion, and metabolism (Figure 2G). This analysis revealed that cluster 1 in CRC5 was significantly enriched in tumor stemness‐associated pathways, whereas cluster 6 showed significant enrichment in drug response and Notch signaling pathways.

In summary, our findings suggest that clusters 1 and 6 play pivotal roles in irinotecan resistance in CRC5. Cluster 1 contributes to resistance through the regulation of coagulation functions and tumor stemness pathways, while cluster 6 facilitates resistance by reprogramming lipid metabolism and activating Notch signaling pathways. These results provide important insights into the molecular mechanisms of irinotecan resistance in CRC.

### 3.4 Pseudotime Analysis

**FIGURE 3:**
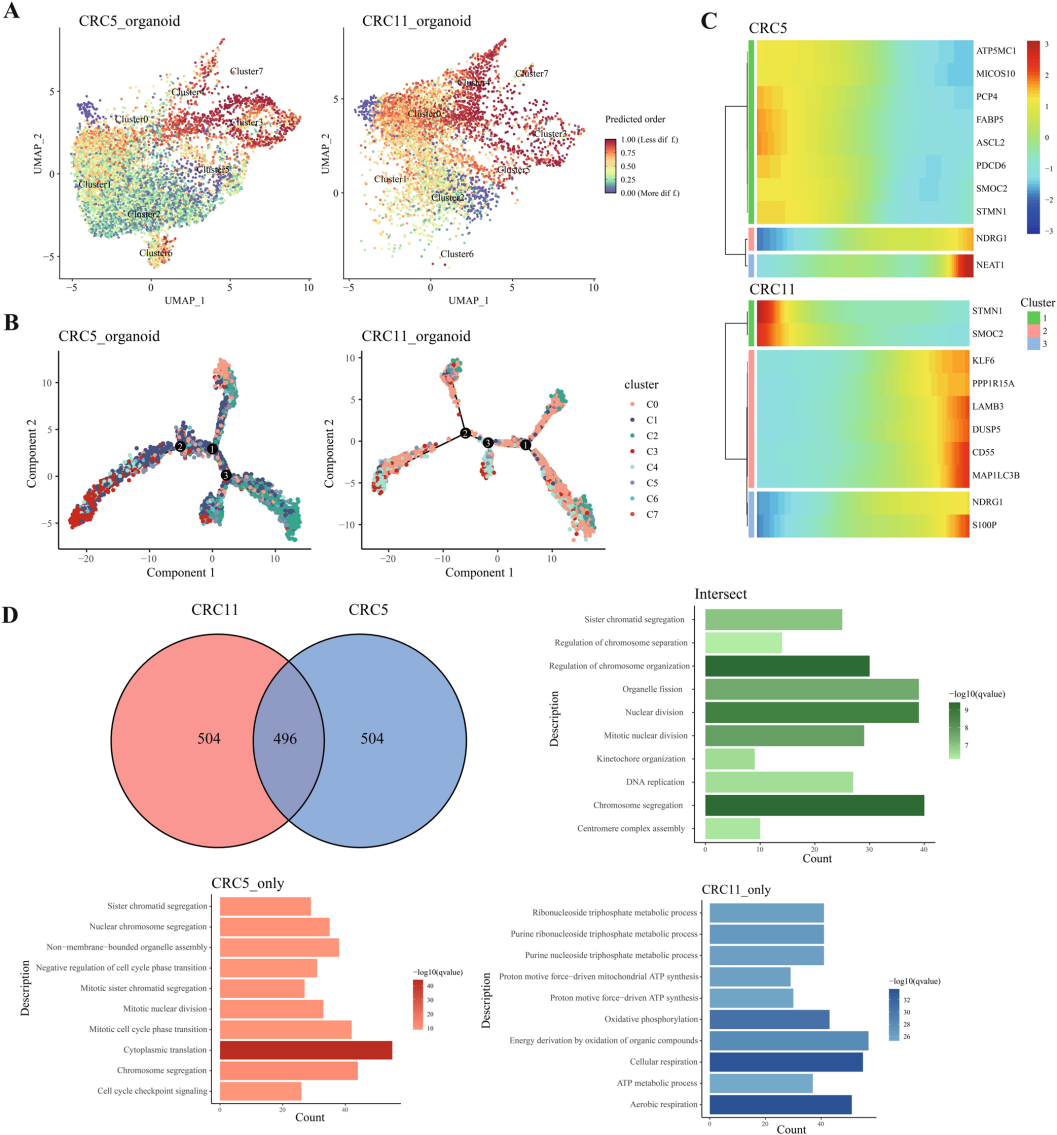
Pseudotime analysis of organoids. (A) Cell differentiation states in the two organoids, where 0.0 (blue) represents a highly differentiated state and 1.0 (red) indicates a less differentiated state. (B) Pseudotime analysis of cells transitioning from low to high differentiation status. (C) Top 10 genes associated with pseudotime in both organoids. (D) Venn diagram and enrichment analysis of the top 1000 pseudotime‐related genes in both organoids.

To investigate the differentiation trajectory of cells within the organoids and its potential association with irinotecan resistance, we performed pseudotime analysis on the CRC5 and CRC11 organoids using the R package CytoTRACE (Figure 3A). The analysis revealed that cluster 1, which exhibits stemness characteristics, is widely distributed across various differentiation stages in CRC5 (Figure 3B), suggesting that cluster 1 may play a crucial role in the maintenance of tumor stem cells and the development of resistance. By constructing a trajectory from low to high differentiation (from left to right), we identified key pseudotime genes that significantly contribute to the trajectory and listed the top 10 genes (depicted in a gradient from light to dark blue, indicating increasing contribution) (Figure 3C). To independently validate the biological relevance of these pseudotime‐dependent genes, we analyzed their expression in GSE145356. Reassuringly, the majority of the top pseudotime genes exhibited significant differential expression in this independent dataset (Figure S1E), thereby robustly corroborating our pseudotime trajectory predictions.

Further functional enrichment analysis of the top 1000 pseudotime‐related genes revealed several pathways, including DNA replication and regulation of chromosome organization. CRC11‐specific GO pathways included oxidative phosphorylation, ATP synthesis, and metabolic processes, while CRC5 enriched pathways involved cytoplasmic translation and regulation of the cell cycle (Figure 3D). These findings indicate significant differences between CRC5 and CRC11 in terms of cellular differentiation trajectories and functional pathways. The widespread distribution of cluster 1 in CRC5, along with its associated GO pathways, may contribute to irinotecan resistance by maintaining tumor stem cell characteristics and proliferative capacity. In contrast, the enrichment of oxidative phosphorylation and ATP metabolic pathways in CRC11 may underlie its sensitivity to irinotecan. These results provide valuable insights into the potential mechanisms of irinotecan resistance in CRC and lay the theoretical foundation for future targeted therapeutic research.

### 3.5 Cell–Cell Communication

**FIGURE 4:**
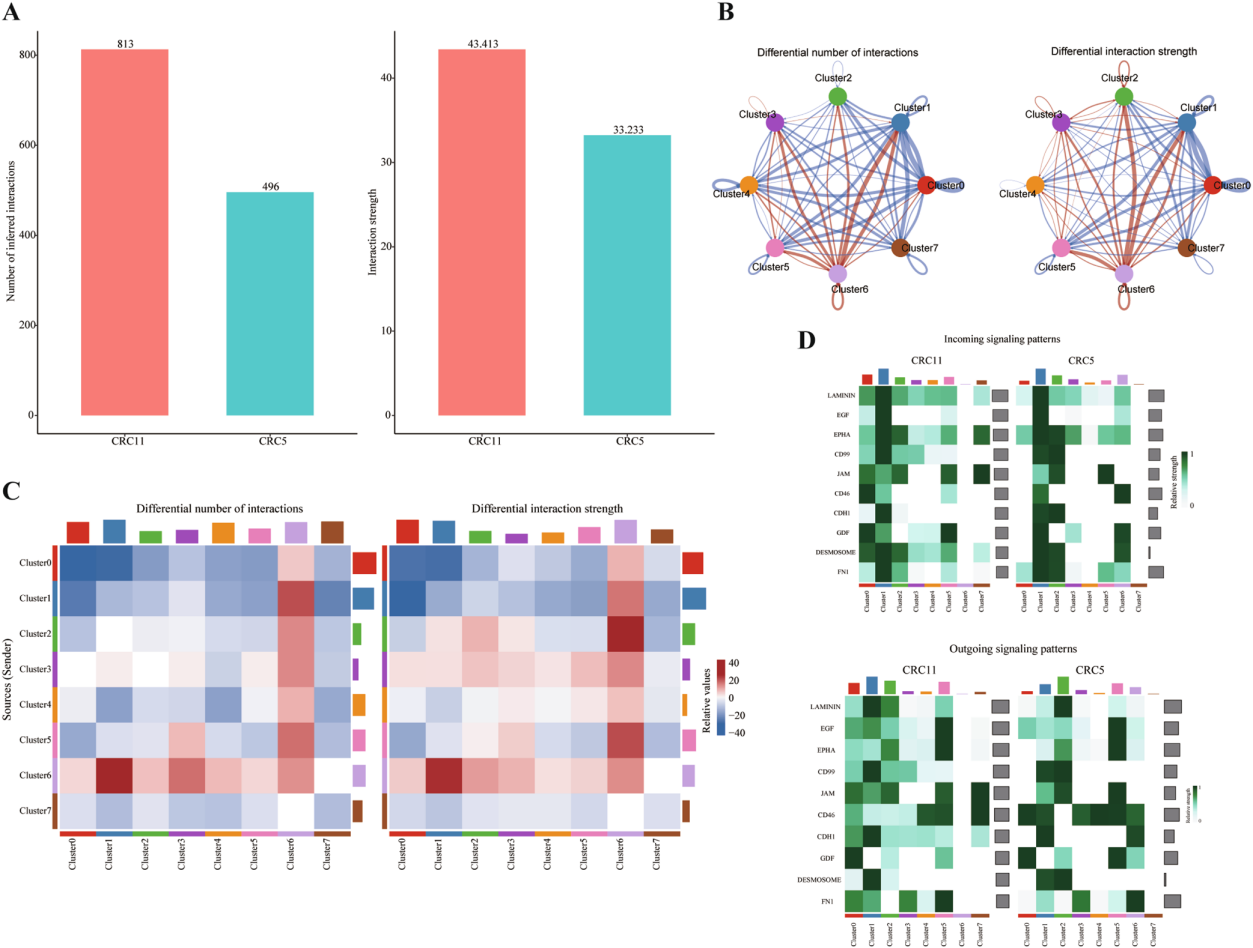
Cell communication analysis of organoids. (A) Differences in the number and intensity of cell–cell communication between the two organoids. (B) Differences in the number and intensity of cell–cell communication between clusters in the two organoids. Red lines indicate increased communication, while blue lines represent decreased communication. (C) Heatmap of the number and intensity of cell–cell communications between clusters in the two organoids. Red blocks indicate increased communication, while blue blocks represent decreased communication. The bar graph at the top represents the total signal received by each cluster, and the bar graph on the right represents the total signal sent by each cluster. (D) The top 10 signaling pathways contributing to communication in both organoids. The color blocks represent relative signal intensity, with the top bar graph showing the total signal intensity received by each cluster and the right bar graph showing the total signal intensity of each signaling pathway.

To investigate the role of cell–cell communication in irinotecan resistance in CRC, we compared the total number and interaction intensity of cell–cell communication networks between CRC11 and CRC5 organoids (Figure 4A). Overall, the cell communication network in CRC5 exhibited weaker interaction strength, suggesting that intercellular communication may be suppressed in this model. To further explore the changes in communication between different cell clusters, we examined the differences in the number and strength of communications among individual clusters within CRC11 and CRC5 (Figure 4B,C). Compared to CRC11, CRC5 cluster 6 showed a significant enhancement in cell–cell communication, indicating that it may play a crucial role in resistance development by strengthening intercellular interactions. Additionally, we analyzed the contributions of input/output signaling pathways in both organoids (Figure 4D), identifying the top 10 signaling pathways with the highest contributions. These pathways may provide valuable insights into the underlying mechanisms of irinotecan resistance in CRC.

**FIGURE 5:**
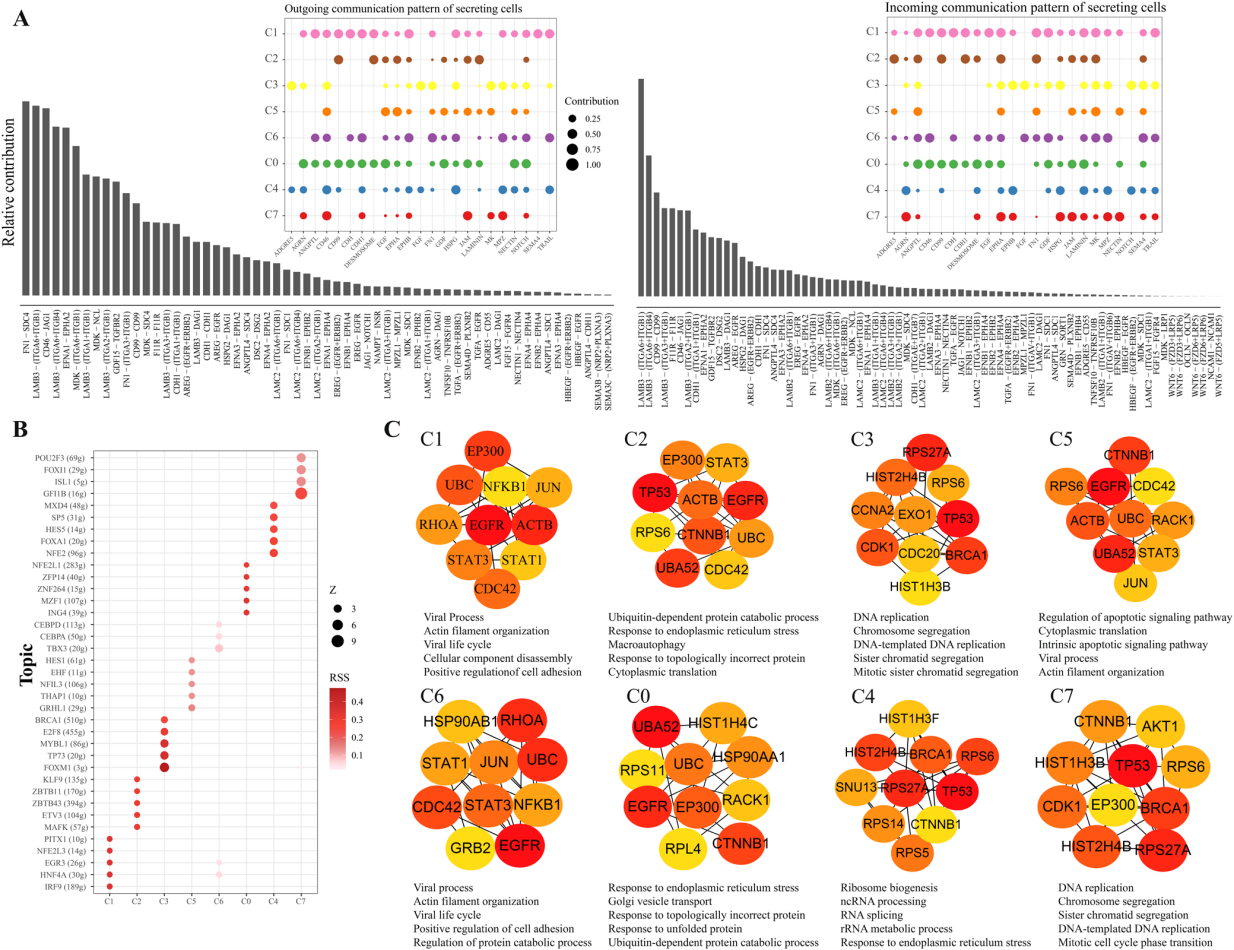
Cell–cell interactions in organoids. (A) Contribution of cluster‐specific signaling pathways and ligand‐receptor pairs between CRC11 and CRC5 as determined by CellChat analysis. (B) Top transcription factors for each cluster identified by SCENIC, where the depth of color and size of the points represent the *z*‐score and RSS score, respectively. (C) Molecular cluster‐specific gene regulatory networks for eight clusters, as analyzed by the SCENIC package, based on gene expression profiles and transcription factor binding motif information.

We analyzed the ligand‐receptor interactions and cell‐specific signaling pathways in organoids to elucidate the potential mechanisms underlying irinotecan resistance. Through analysis of a ligand‐receptor database, we identified key ligand‐receptor pairs involved in intercellular communication (Figure 5A). The ECM, EGFR, and Wnt signaling pathways were significantly upregulated. These included LAMB3 and its receptors ITGA6 + ITGB1, ITGA6 + ITGB4, ITGA3 + ITGB1, ITGA2 + ITGB1; EGFR and its ligands HBEGF and AREG; and WNT6 and its receptors FZD5 + LRP5, FZD6 + LRP6, FZD6 + LRP5, FZD3 + LRP6, and FZD3 + LRP5. Activation of these pathways may enhance tumor cells' stemness and proliferative capacity, thereby contributing to irinotecan resistance. Additionally, the Notch and SEMA signaling pathways were significantly upregulated, including JAG1 and its receptor NOTCH1, SEMA3C and its receptor NRP2 + PLXNA3. Activation of these pathways may influence cell migration and invasion, thus playing a role in irinotecan resistance. To pinpoint the most therapeutically relevant interactions, we further scrutinized the communication patterns between key clusters. This refined analysis revealed that LAMB3‐integrin interactions were not only highly active globally but also constituted dominant signaling pathways from the Clusters 2 to Cluster 1 and Cluster 6 (Figure S2).

To further dissect the resistance mechanisms, we employed SCENIC (Single‐Cell Regulatory Network Inference and Clustering) to analyze transcriptional regulators in each cluster. The results revealed distinct transcriptional regulators for each cluster (Figure 5B). For example, cluster 1 exhibited specific transcriptional regulators such as PITX1, NFE2L3, and IRF9, with NFE2L3 modulating tumor stemness via the Wnt pathway. Cluster 6 exhibited specific transcriptional regulators, including CEBPD, CEBPA, and HNF4A, which play key roles in regulating cellular lipid metabolism. We sought external validation for the clinical and functional relevance of these key transcription factors. Interrogation of the TCGA CRC cohort revealed that high expression levels of NFE2L3 and CEBPA were significantly associated with a shorter disease‐free interval (Figure S3A). Moreover, the expression of NFE2L3 and CEBPA showed strong positive correlations with their respective predicted target genes from the SCENIC analysis (Figure S3B,C), providing compelling evidence for the integrity of the inferred regulatory networks.

To gain deeper insights into intercellular interactions, we constructed molecular cluster‐specific GRNs for each cluster based on gene expression profiles and transcription factor data (Figure 5C). Analysis of hub genes and associated signaling pathways revealed significant enrichment of pathways such as Viral Process, Actin Filament Organization, Viral Life Cycle, and Positive Regulation of Cell Adhesion in clusters 1 and 6.

### 3.6 Functional Validation of Key Pathways in SN‐38 Resistance

**FIGURE 6:**
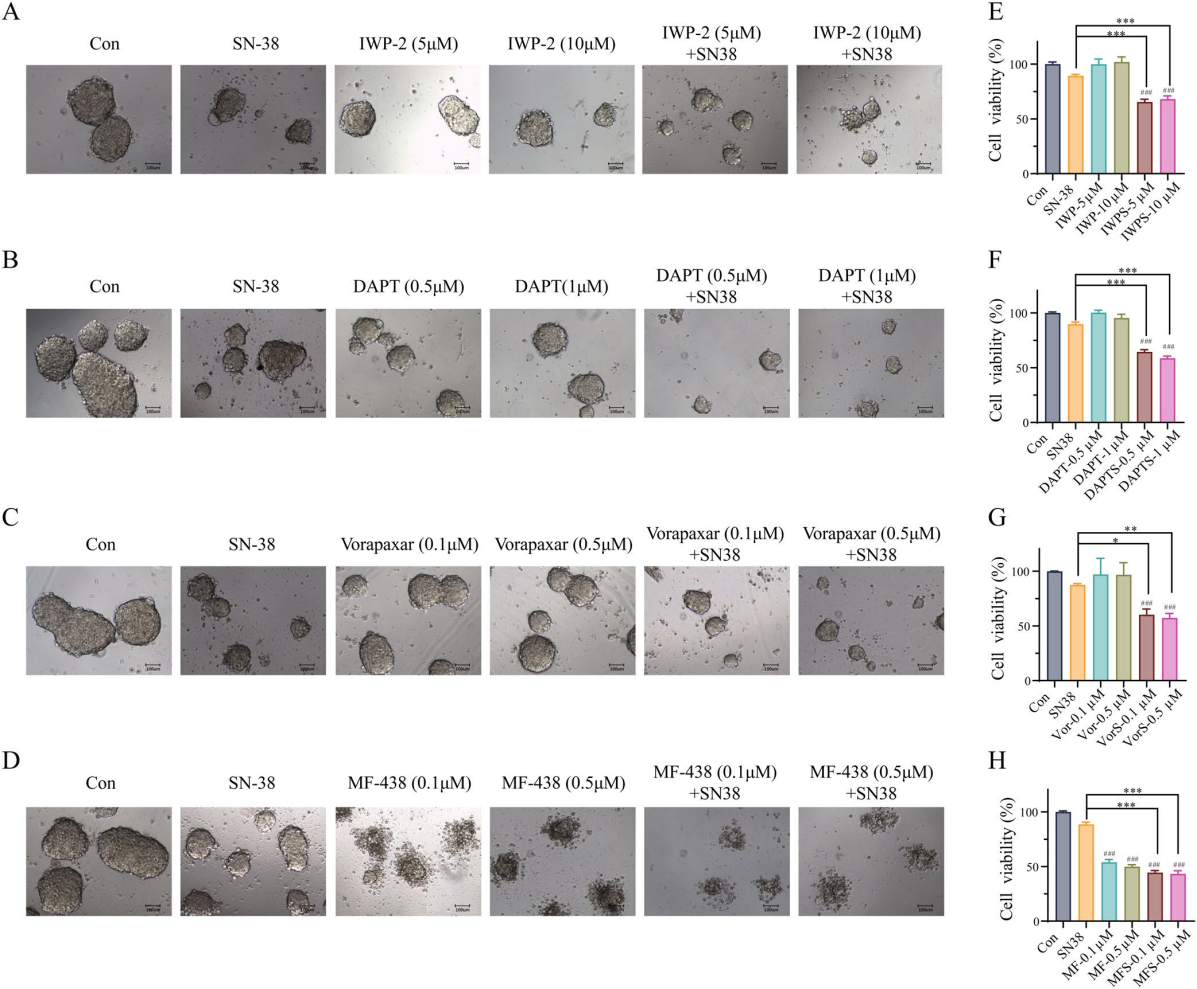
Inhibitors of various pathways reversed the resistance of CRC5 to SN‐38. (A–D) Representative raw images of morphology of colorectal cancer PDOs treated with SN‐38 (5 μM), IWP‐2 (5 μM and 10 μM), DAPT (0.5 μM and 1 μM) (B), Vorapaxar (0.1 μM and 0.5 μM) (C), MF‐438 (0.1 μM and 0.5 μM) (D) or in combination for 3 days. (E–H) Cell viability in colorectal cancer PDOs detected by CellTiter‐Blue Reagent. The data in the graph were analyzed by one‐way ANOVA, followed by Tukey's HSD test for pairwise comparisons. Significance levels are indicated as *P < 0.05, **P < 0.01, ***P < 0.001.

To investigate whether the Wnt, Notch, lipid metabolism, and coagulation pathways play a causative role in SN‐38 resistance, we performed functional validation experiments. We treated the CRC5 organoid model with specific pathway inhibitors—IWP‐2 (a Wnt pathway inhibitor), DAPT (a Notch pathway inhibitor), MF‐438 (a SCD1‐mediated lipid metabolism inhibitor), and Vorapaxar (a coagulation pathway inhibitor)—in combination with 5 μM SN‐38. As shown in Figure 6, the co‐treatment of SN‐38 with any of these inhibitors resulted in a significant increase in the growth inhibition rate of CRC5 organoids compared to SN‐38 treatment alone. These functional findings demonstrate that pharmacological inhibition of these pathways can effectively sensitize CRC5 to SN‐38, indicating that these pathways are not merely correlative but functionally contribute to the irinotecan resistance mechanism.

In summary, our integrated analysis reveals that the distinct irinotecan resistance phenotype of CRC5 is underpinned by systematic differences in ligand‐receptor interactions, transcriptional regulators, and GRNs compared to the sensitive CRC11 model. The resistant organoid is characterized by Cluster 1 and Cluster 6, which exhibited marked specificity in CRC5 and were independently validated in an external cohort. These clusters likely drive resistance through the coordinated activity of key signaling pathways: Cluster 1 via Wnt signaling and extracellular matrix remodeling, and Cluster 6 through lipid metabolic reprogramming and Notch signaling. Furthermore, cell communication analysis nominated the LAMB3‐integrin axis as a prioritized target within this network. We propose that the key molecular features of these clusters collectively enhance irinotecan resistance by modulating core cellular processes including stemness maintenance, survival signaling, and adaptive drug responses. These findings not only provide a mechanistic framework for understanding irinotecan resistance but also unveil novel candidate targets for the development of therapeutic strategies aimed at overcoming treatment failure in CRC.

## 4 Discussion

Irinotecan is one of the key agents in the treatment of metastatic CRC, with survival outcomes significantly better than those achieved with 5‐FU and leucovorin chemotherapy. However, the development of resistance, occurring in approximately 50%–60% of patients, poses a major clinical challenge. This resistance is a multifaceted phenomenon, driven by genetic mutations, alterations in drug metabolism, and dynamic adaptations within the tumor microenvironment. Utilizing PDO models combined with single‐cell RNA sequencing, this study provides a high‐resolution dissection of the cellular and molecular landscape of irinotecan resistance.

Our analysis identified several cell clusters with altered abundance in the resistant CRC5 organoid. We focused our mechanistic investigation on Cluster 1 (MARCKSL1+) and Cluster 6 (AKR1C3+), as they demonstrated the most dramatic and specific enrichment. Cluster 1 exhibited a greater than 10‐fold increase, while Cluster 6 was almost exclusively present in the resistant model. These clusters appear to play pivotal roles in developing resistance through distinct pathways. Our discoveries not only elucidate the adaptive changes of tumor cells under drug pressure but also provide new clues for future targeted therapies.

Cluster 1 was significantly enriched in pathways related to coagulation function. Research has demonstrated that coagulation factors, such as thrombin, enhance the stemness and invasiveness of cancer stem cells by activating the protease‐activated receptor (PAR) signaling pathway [38]. Moreover, the hypercoagulable state associated with tumors is closely linked to metastasis and recurrence [39], and coagulation factor X can promote tumor growth by inhibiting antitumor immune responses [40]. Therefore, we hypothesize that cluster 1 may enhance cancer stem cells' stemness and drug resistance by regulating coagulation factors to activate the PAR signaling pathway, which could be one of the mechanisms underlying resistance in CRC5.

In Cluster 6 of CRC5, we observed abnormally active cell communication and were significantly enriched in lipid metabolism‐related pathways. Studies have shown that the key lipid metabolism enzyme SCD1 maintains the self‐renewal capacity of cancer stem cells by modulating the Wnt/β‐catenin and AMPK signaling pathways, thereby enhancing tumor resistance to chemotherapeutic drugs [41, 42, 43]. Gene‐set variation analysis (GSVA) revealed significant activation of the Notch signaling pathway in cluster 6. Notch signaling promotes the formation of drug resistance by regulating downstream pathways such as PI3K/AKT and NF‐κB [44]. Therefore, we speculate that cluster 6 may enhance drug resistance in cancer stem cells through the synergistic action of lipid metabolism reprogramming and Notch signaling, representing another important mechanism contributing to resistance in CRC5.

A pivotal finding from our cell–cell communication analysis was the identification of the LAMB3‐integrin axis as a central and robust communication module, with LAMB3 engaging in multiple high‐probability interactions with various integrins (e.g., ITGA6 + ITGB4, ITGA3 + ITGB1). LAMB3, a key basement membrane component, has been implicated in tumor progression and chemoresistance in other cancers by enhancing integrin‐mediated survival signals and ECM remodeling [45]. Therefore, we propose that targeting the LAMB3‐integrin interface represents a promising and highly specific strategy for disrupting the core circuitry of irinotecan resistance, worthy of prioritization in future functional studies.

It is crucial to acknowledge the limitations of our study. The primary limitation is the relatively small cohort of one sensitive and one resistant PDO, which, while providing deep mechanistic insights, limits the generalizability of our findings. Furthermore, potential confounding factors, such as differences in anatomical subsite (rectum vs. sigmoid colon) and patient age, could have influenced the transcriptomic profiles. Our findings are also derived from Microsatellite Stable (MSS) tumors, and their direct applicability to Microsatellite Instability‐High (MSI‐H) CRC, which has a distinct pathogenesis and treatment paradigm centered on immunotherapy, remains to be established. Moreover, we have provided external validation through public datasets. Interrogation of TCGA data revealed that high expression of key transcription factors identified in our SCENIC analysis (NFE2L3 in Cluster 1 and CEBPA in Cluster 6) was significantly associated with a shorter disease‐free interval, underscoring their clinical relevance.

In conclusion, we propose a model wherein irinotecan resistance in CRC5 is driven by the cooperative action of Clusters 1 and 6. Cluster 1 maintains stem cell characteristics through Wnt signaling and coagulation/ECM pathways, while Cluster 6 enhances survival adaptation via lipid metabolism and Notch signaling. The LAMB3‐integrin axis emerges as a critical mediator of cellular crosstalk within this resistant ecosystem. Our findings uncover unique cellular states and molecular networks in irinotecan‐resistant CRC, providing a valuable resource and novel candidate targets for future research. These insights could offer novel directions for developing personalized treatment strategies. Future work will focus on validating these mechanisms in larger, matched cohorts, including MSI‐H models, ultimately aiming to translate these findings into improved therapeutic outcomes for CRC patients.

## Author Contributions

**Yi Pan:** data curation, formal analysis, investigation, methodology, software, writing – original draft, visualization, writing – review and editing. **Lin Chen:** funding acquisition, validation, visualization, writing – original draft, writing – review and editing. **Yuqing Hu:** investigation, funding acquisition. **Jie Chang:** data curation. **Xifeng Xu:** investigation, resources. **ShuoChen Xu:** data curation, resources. **YiWen Li:** investigation. **Jinlin Du:** conceptualization, project administration. **JianPing Wang:** funding acquisition, supervision. **Wenxia Xu:** funding acquisition, conceptualization, supervision.

## Funding

This work was supported by the Social Development Key Project of Jinhua Science and Technology Planning Project, China (2026‐3‐039, 2021‐3‐039, 2023‐3‐111); Chinese medicine science and technology project of Zhejiang Province, China (2024ZL1183); Research Fund Project of Zhejiang Medicine and Health Science and Technology Program, China (2024KY502); Public Welfare Technology Research Program of Zhejiang Province, China (LTGY23H160025) and Emerging Medical Talent of Zhejiang Provincial Health Commission.

## Ethics Statement

The research involving human participants adhered to the principles of the Declaration of Helsinki. The study protocol received formal review and approval from the Ethics Committee of Affiliated Jinhua Hospital, Zhejiang University School of Medicine, under the approval number (Research) 2021—Ethical Review—257. Prior to their participation, all subjects provided written informed consent. All personal data collected were handled confidentially and anonymized during analysis to protect participant privacy.

## Conflicts of Interest

The authors declare no conflicts of interest.

## Supporting information


**Figures S1–S3:** cam471550‐sup‐0001‐FiguresS1‐S3.docx.

## Data Availability

The data that support the findings of this study are openly available in GEO at https://www.ncbi.nlm.nih.gov/geo/.
