# Supplementary material for: Colorectal Cancer Organoid Model Reveals the Mechanisms of Irinotecan Resistance at Single‐Cell Resolution
Source: Cancer Med. 2026 Feb 12;15(2):e71550. doi: 10.1002/cam4.71550 (PMC12900261; doi:10.1002/cam4.71550)
Supplement: Supplementary file 1 — Figures S1–S3: cam471550‐sup‐0001‐FiguresS1‐S3.docx. [file CAM4-15-e71550-s001.docx]

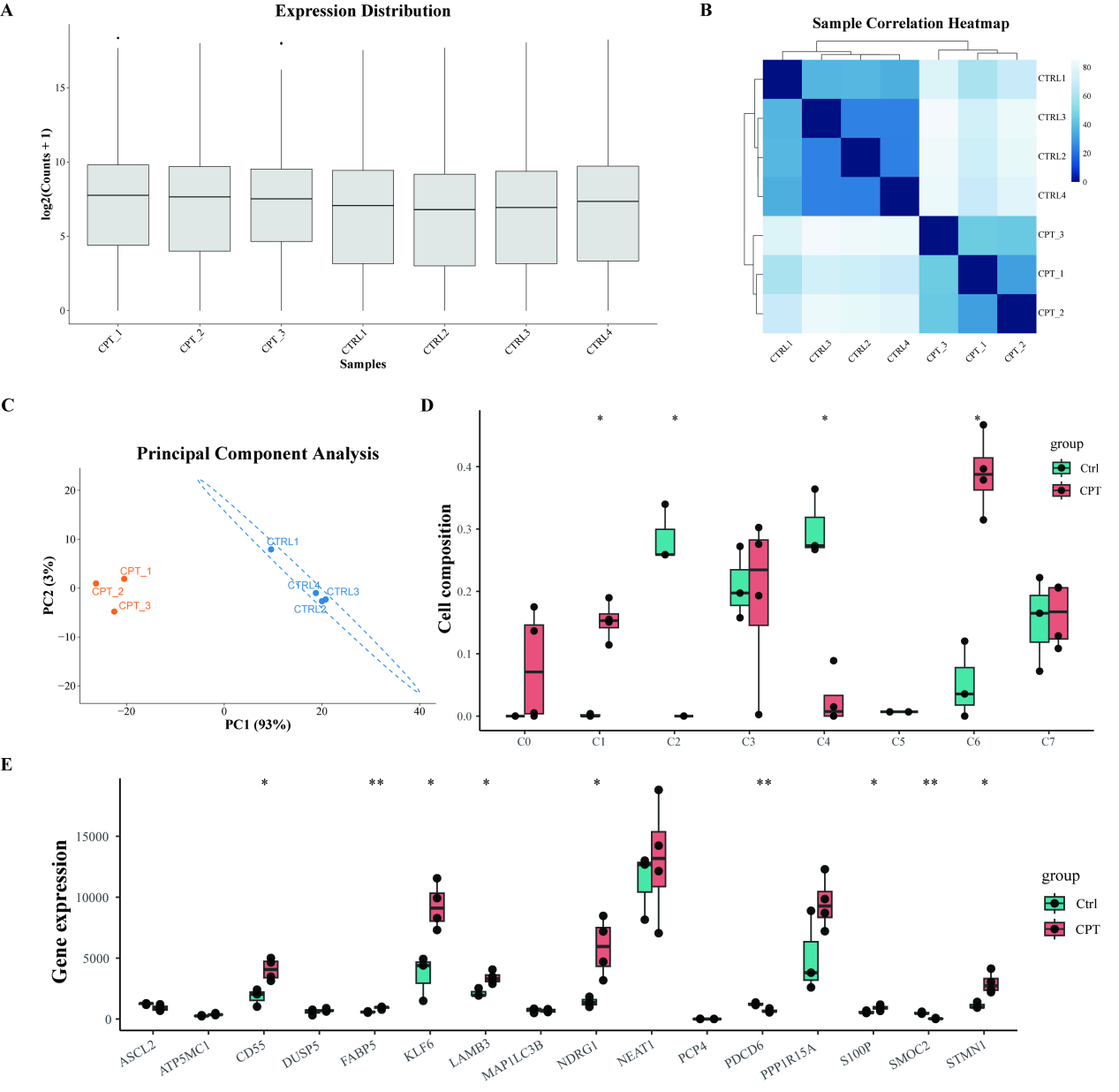


**Supplementary Figure 1. Analysis of the GSE145356 cohort. (**A) Boxplot illustrating the distribution of gene expression levels across all samples. (B) Heatmap depicting the gene expression profiles of samples. (C) Principal component analysis plot showing the separation between the two experimental groups. (D) Boxplot representing the deconvolution results of single-cell clusters. (E) Boxplot displaying expression levels of pseudotime-dependent genes.


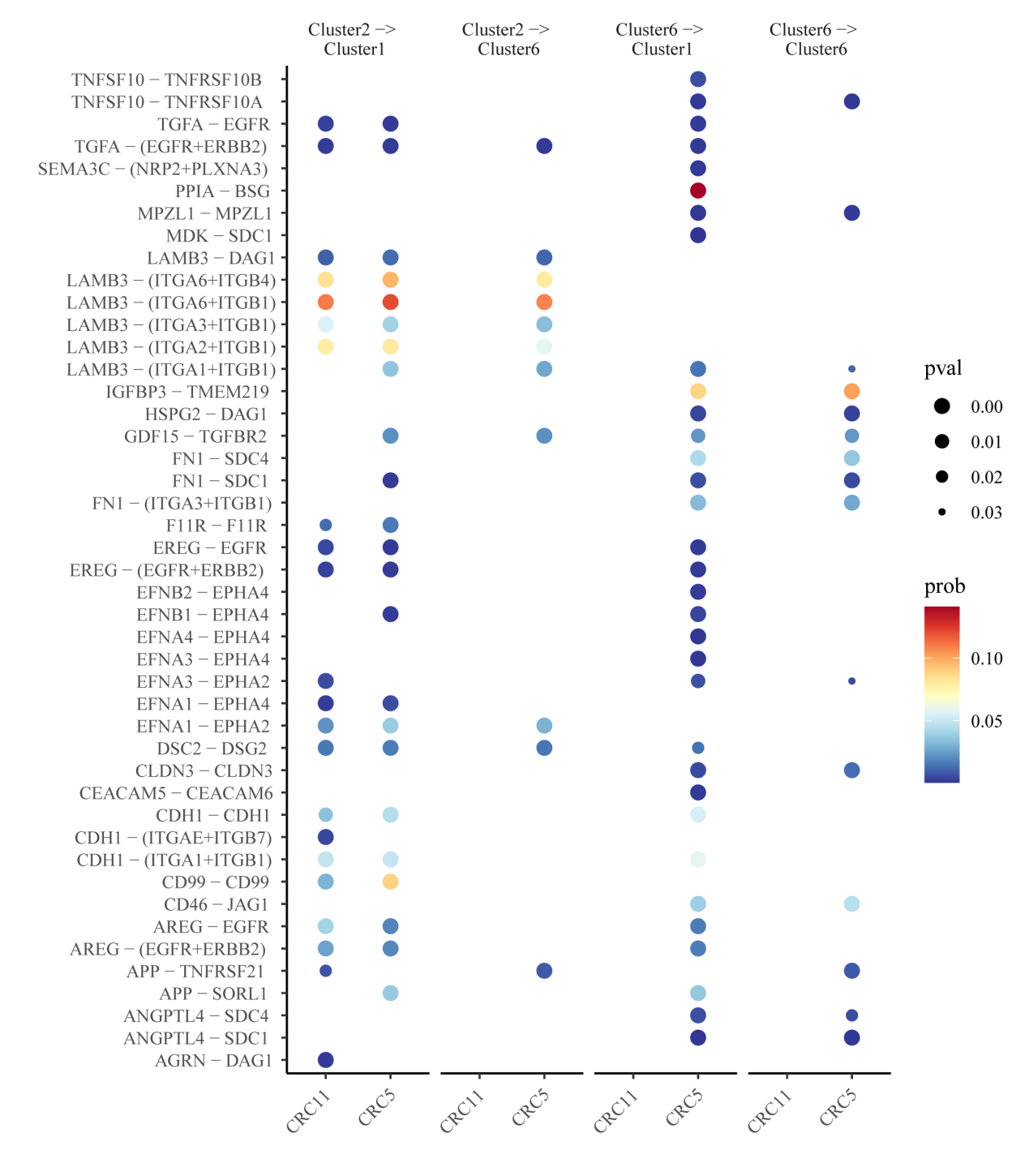


**Supplementary Figure 2. Bubble plot of cell-cell communication analysis.**

**
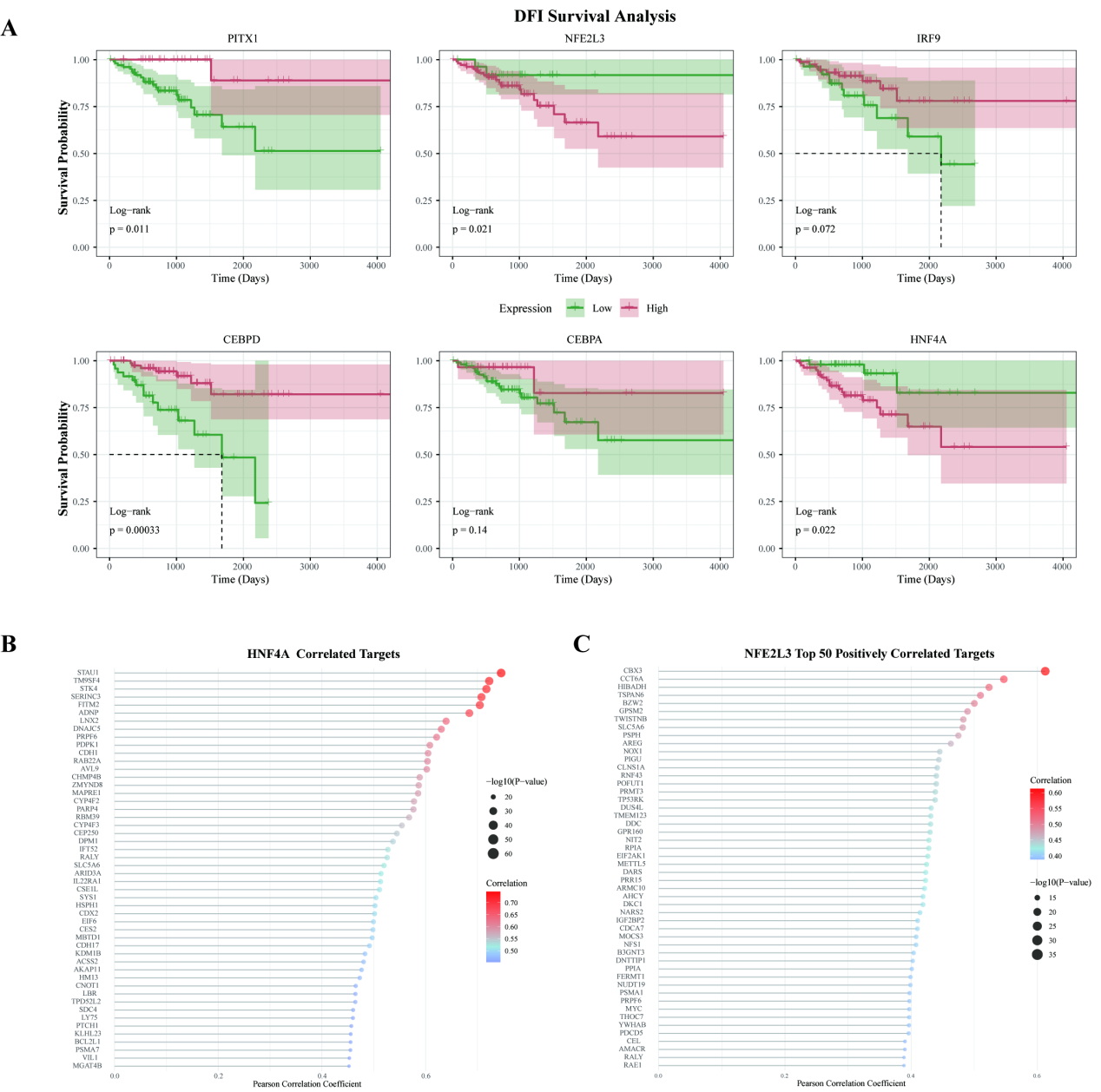
**

**Supplementary Figure 3. Analysis of TCGA Data.** (A) Kaplan-Meier curves depicting the disease-free interval (DFI) survival of patients stratified by the expression levels of six transcription factors. (B) Lollipop plots displaying the correlation between the expression of transcription factor HNF4A and their respective target genes. (C) Lollipop plots displaying the correlation between the expression of transcription factor NFE2L3 and their respective target genes.
